# Bio-Based Epoxy Adhesives Reinforced with Recycled Fillers

**DOI:** 10.3390/polym17222975

**Published:** 2025-11-07

**Authors:** Alberto Cellai, Lorenzo Pezzana, Valentina Casalegno, Milena Salvo, Marco Sangermano

**Affiliations:** Dipartimento di Scienza Applicata e Tecnologia, Politecnico di Torino, C.so Duca degli Abruzzi 24, 10129 Torino, Italy; alberto.cellai@polito.it (A.C.); valentina.casalegno@polito.it (V.C.); milena.salvo@polito.it (M.S.)

**Keywords:** biobased, recycling, circular economy, sustainability, adhesives, thermal curing

## Abstract

This study explores the potential of a bio-based thermosetting adhesive system incorporating recycled fillers to enhance structural bonding applications while promoting sustainability. Diglycidylether of vanillyl alcohol (DGEVA) was selected as the resin matrix due to its favorable thermomechanical properties and low moisture absorption. To improve mechanical performance and support circular economy principles, recycled carbon fibers (RCFs) and mineral wool (MW) were integrated into the adhesive formulation in varying proportions (10, 30, and 50 phr). A cationic thermal initiator, ytterbium (III) trifluoromethanesulfonate (YTT), was used to permit polymerization. Comprehensive characterization was performed to assess the curing behavior, thermal stability, and mechanical performance of the adhesive. FTIR spectroscopy monitored the polymerization process, while DSC and dynamic DSC provided insights into reaction kinetics, including activation energy, and curing rates. The mechanical and thermomechanical properties were evaluated using dynamic mechanical thermal analysis (DMTA) and shear lap testing on bonded joints. Additionally, SEM imaging was employed to examine fillers’ morphology and joint interfaces. The results indicated that increasing filler content slowed polymerization and raised activation energy but still permitted high conversion rates. Both RCF- and MW-containing formulations exhibited improved stiffness and adhesion strength, particularly in CMC joints. These findings suggest that DGEVA-based adhesives reinforced with recycled fillers offer a viable and sustainable alternative for structural bonding, contributing to waste valorization and green material development in engineering applications.

## 1. Introduction

In recent decades, the use of bio-based polymers has expanded significantly across industries such as aerospace and automotive [1]. These materials, which serve as bulk components, matrices, and adhesives, have garnered increasing attention due to their potential to reduce dependency on fossil fuels. While bio-based polymers still represent a small share of the global plastics market, the growth of the bioeconomy has spurred their adoption in diverse applications [2]. Researchers are particularly drawn to natural resource-based polymers for their environmental benefits and potential to support sustainable development [3,4].

Despite this growing interest, the synthesis and production of bio-based structural adhesives remain with lack of exploration. A key challenge lies in the mechanical degradation of these adhesives under humid conditions, which limits their performance in demanding applications [5,6]. Currently, most bio-based adhesives are designed for medical purposes, prioritizing biodegradability and biocompatibility [7,8,9]. Soy-based adhesives, for example, have shown promise in wood applications, while other materials such as lignin, starch, cellulose, and natural oils have been investigated for their potential to reinforce bio-based adhesives [10]. Efforts to synthesize adhesives from proteins have also been reported, but these materials often fall short of being fully bio-based or meeting the necessary mechanical standards [11,12,13].

One promising development is the use of vanillyl alcohol as a building block for bio-based monomers. The cationic polymerization of its diglycidyl ether derivative (DGEVA) yields materials with exceptional thermomechanical properties and low moisture absorption, making them suitable for advanced applications, including aerospace and space industries [14,15].

However, like other thermosetting resins, bio-based adhesives based on DGEVA are not reusable after curing [16]. Epoxy resins typically end up as waste after their service life, with limited opportunities for direct reuse [17]. Most recycling approaches involve pulverizing cured epoxy materials for use as fillers or reinforcements in new structures, such as concrete, composite materials, and construction products [18].

The same path is followed by one of the main fillers used in epoxy resins, carbon fibers. Carbon fiber recycling has emerged as a notable area of focus, given the significant volumes of manufacturing waste and end-of-life products. Although recycled carbon fibers retain most of their mechanical properties, their reduced length limits their reuse to lower-performance applications [19].

Similarly, the recycling of mineral wool, widely used in the construction industry for insulation and fire protection, represents another significant opportunity [20]. Current methods repurpose mineral wool waste in cement-based composites, ceramics, or wood fiber products [21]. However, there is growing interest in incorporating both recycled carbon fibers and mineral wool waste into polymer composites to address the rising demand for such materials. This approach not only reduces landfill waste but also conserves natural resources by substituting virgin raw materials with recycled components [22,23,24].

This study investigates the feasibility of developing a structural bio-based adhesive utilizing DGEVA as a base material, reinforced with recycled carbon fibers and mineral wool waste derived mainly from demolished buildings. The primary objective is to address dual challenges: enhancing the performance of bio-based adhesives and advancing the principles of the circular economy by integrating industrial and construction waste into high-value applications. By incorporating recycled reinforcements, this research aims to improve the mechanical properties of DGEVA-based adhesives while preserving their bio-based nature and ensuring their suitability for structural use. Furthermore, the study evaluates the potential environmental benefits and practical applications of such adhesives in industries that demand high-performance encompassed by sustainable materials.

## 2. Materials and Methods

### 2.1. Materials

**Figure 1:**
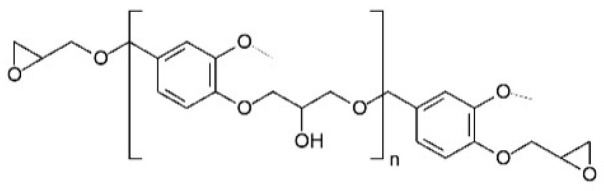
Diglycidylether of vanillyl alcohol chemical structure.

All the samples were prepared using as the polymeric matrix Diglycidylether of vanillyl alcohol (DGEVA), procured from Specific Polymers (Castries, France). The general chemical structure of DGEVA is visible in Figure 1 [25].

**Figure 2:**
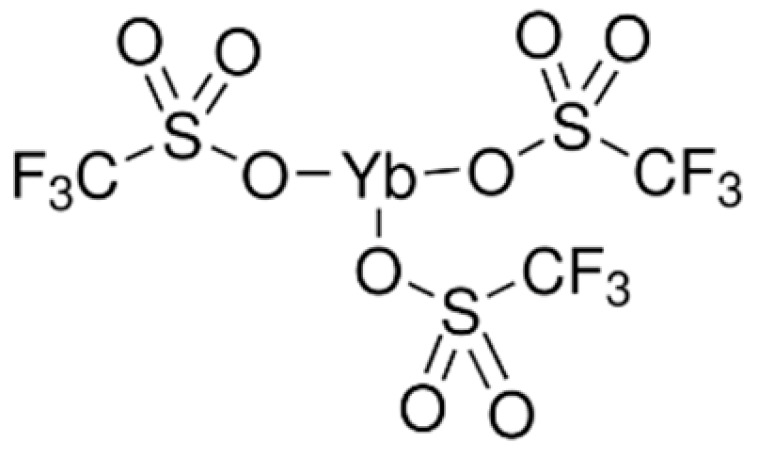
Ytterbium (III) trifluoromethanesulfonate chemical structure.

As a cationic thermal initiator, Ytterbium (III) trifluoromethanesulfonate (YTT) was used, supplied by Sigma Aldrich (Milan, Italy). Its chemical structure is reported in Figure 2 [26].

**Figure 3:**
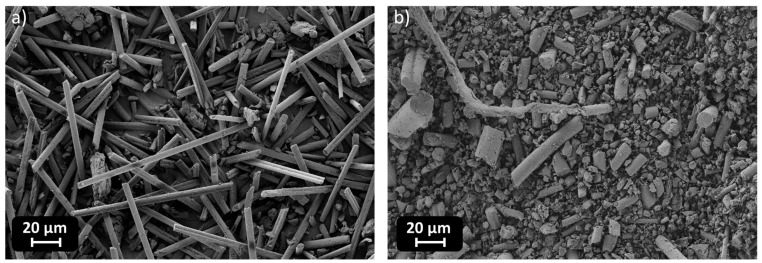
SEM images of RCF (**a**) and MW (**b**) as free fillers.

Additionally, different types of fillers were introduced in different amounts: recycled carbon fibers (RCFs), with a length of 0.2 mm, and recycled mineral wool (MW). RCFs were recovered from dismissed composites while MW is obtained from insulation panels in buildings. SEM images of the free fillers are visible in Figure 3.

### 2.2. Formulations Preparation

A total of seven formulations have been analyzed, each with different filler content. Table 1 reports the formulations studied.

In each formulation, 2 phr of cationic thermal initiator, the YTT, was added.

### 2.3. Characterization of the Formulations

All the formulations were analyzed in their liquid and solid form by means of the following methods.

#### 2.3.1. FTIR

IR spectroscopy was used to measure the conversion of the resin upon thermal curing. A lowering of the peak characteristic of epoxies after curing is expected. The conversion was calculated using Equation (1) [25,27,28,29,30]:

(1) $$\;Conversion\;(\%)=\;\frac{(\frac{A_{functional}}{A_{reference}}{)}_{t=0}-(\frac{A_{functional}}{A_{reference}}{)}_{t}}{(\frac{A_{functional}}{A_{reference}}{)}_{t=0}}\cdot 100$$

where *A*_functional_ is the peak area of the functional group under investigation during the test, specifically the epoxy groups (the peak from the epoxide asymmetric C-O-C stretch, that appears from 950 to 810 cm^−1^, and the symmetric C-O-C stretch, where the two C-O bonds stretch while the C-C bond contracts, found from 880 to 750 cm^−1^), and *A*_reference_ is the area of the peak used as a reference, the carbonyl one in this case, centered at around 1780 and 1680 cm^−1^ [31], with t = 0 and t, the pre- and post-thermal energy exposure.

The Thermo ScientificTM NicoletTM iS50 FT-IR spectrometer (Milan, Italy) and OmnicTMSpectra software were used to conduct this analysis; the oven used to perform the thermal curing is ArgoLab TCN30 Plus. The formulations were deposited on a silicon substrate with a spreader bar, in the form of films approximately 12 μm thick. Subsequently, the IR spectrum of the test sample was recorded prior to thermal exposure (pre-curing, t = 0 min). After that, each film was inserted in the oven at 150 °C for 2 h and 180 °C for 2 h. After the curing step, the IR spectrum of the sample was again recorded. In a second period, the conversion percentage of the samples is calculated by using Equation (1).

#### 2.3.2. Dynamic DSC and DSC

The dynamic DSC and DSC analyses were performed using a Mettler TOLEDO DSC-1 (Milan, Italy) instrument under a nitrogen atmosphere, with a flow rate maintained at 40 mL/min. Three samples of each formulation were analyzed to calculate a weighted average while considering the standard deviation of the obtained values.

For the dynamic DSC tests, 5–10 mg of liquid resin was placed in an aluminum crucible with a capacity of 40 μL, while an empty crucible served as the reference. The heating programs were conducted in the temperature range of 25–300 °C, with ramp rates set at 2, 5, 10, and 20 K/min. Specific heat flow curves [W/g] were recorded as a function of time [s]. The peak height (T_peak_) is directly related to the polymerization rate, with higher values indicating faster polymerization kinetics. The enthalpy change (ΔH) was determined by integrating the area under the heat flow curve.

To evaluate the thermodynamic properties of the curing process, dynamic DSC experiments were conducted and analyzed using the Kissinger method. This approach allows us to determine the apparent activation energy (E_a_) by relating the peak temperature (T_p_) observed during the exothermic curing reaction to the heating rate (ϕ). Specifically, the Kissinger equation is given by (2) [32]

(2) $$\ln\left(\frac{\phi }{T_{p}^{2}}\right)=-\frac{E_{a}}{R}\cdot \frac{1}{T_{p}}+c$$

where ϕ is the heating rate in K/min, T_p_ is the peak temperature in Kelvin, E_a_ is the activation energy in J/mol, and R is the universal gas constant (8.314 J/mol·K). The constant c in this equation is further defined by the pre-exponential factor (A) as (3)

(3) $$c=ln(\frac{A\cdot R}{E_{a}})$$

To quantify the progression of the curing reaction, the determination of the degree of conversion, α, can be obtained by comparing the experimentally measured heat release (ΔH_exp_) to the theoretical total heat of curing (ΔH_the_) via the relation (4):

(4) $$\alpha =\frac{\Delta H_{exp}}{\Delta H_{the}}$$

The rate of conversion can then be expressed as (5)

(5) $$\frac{d\alpha }{dt}=k\left(T\right)\cdot f(\alpha )$$

where $k\left(T\right)$ is a temperature-dependent rate coefficient and $f\left(\alpha \right)\;$ represents the reaction model. Assuming an Arrhenius-type behavior, the rate coefficient is described by (6)

(6) $$k\left(T\right)=A\cdot e^{-\frac{E_{a}}{R\cdot T}}$$

Substituting Equation (6) into Equation (5) yields the general kinetic expression for the curing process (7):

(7) $$\frac{d\alpha }{dt}=A\cdot e^{-\frac{E_{a}}{R\cdot T}}\cdot f(\alpha )$$

In addition to the kinetic analysis provided by dynamic DSC, conventional DSC measurements were employed to evaluate the glass transition temperature (Tg) of the cured formulations. The Tg was identified as the temperature region where a distinct change in heat capacity occurs, typically observed as a step or an inflection point in the DSC baseline. For these measurements, a heating rate of 3 K/min was used over a temperature range from 0 to 200 °C.

### 2.4. Sample Manufacturing

The process to manufacture the joints involved the application of the resin on the top of the substrates, made of Ceramic Matrix Composite (CMC) or aluminum (Al), and then covering it with the second piece of metal or composite. CMC/CMC and Al/Al joints have been produced, at least three joints for each formulation. A support of the same thickness of the substrates has been used to create parallel surfaces of the two pieces which are joined. Additionally, before applying the liquid formulations, the surface of the substrates undergoes degreasing by a treatment with acetone in ultrasound bath for 30 min.

**Figure 4:**
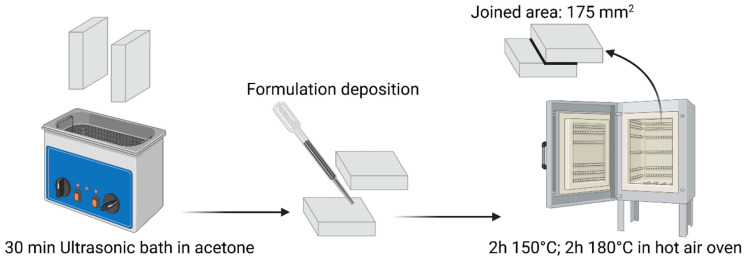
Graphical representation of joint manufacturing.

Once the liquid formulation was correctly deposited and the two substrates correctly positioned, the samples were put in an oven to undergo the curing process. This step consisted of 2 h at 150 °C and 2 h at 180 °C to guarantee a proper curing. The joined area is 175 mm^2^. The scheme of the process is visible in Figure 4.

In addition to joints, DMTA samples were also produced by pouring the liquid formulation into silicon molds and then undergoing the curing process previously explained. Five samples for each formulation have been produced, with dimensions of 18 × 8 × 1 mm^3^.

### 2.5. Characterization of the Cured Samples

#### 2.5.1. DMTA

Dynamic mechanical thermal analysis (DMTA) was performed using a Triton Technology instrument operating in tension mode, where uniaxial stress was applied at a frequency of 1 Hz. The tests were carried out with a temperature range from 25 °C to 225 °C, at a heating rate of 3 K/min. The glass transition temperature (Tg) was determined as the peak of the Tanδ curve.

#### 2.5.2. Shear Lap Test

The tests were conducted on joints in a compressive instrument (MTS QTestTM/10Elite) from MTS System Corporation, with a translation speed of 2 mm^−1^ and a loading cell of 50 kN.

#### 2.5.3. SEM

SEM analyses were conducted to investigate filler dispersion inside the polymeric matrix and adhesion of the formulations with the substrates. More precisely, photos were taken with the Jeol JCM-6000 PLUS (St-Hubert, QC, Canada), for each sample. The analyzed surface was covered with a 10 nm thick platinum layer.

## 3. Results Discussion

### 3.1. FTIR

**Figure 5:**
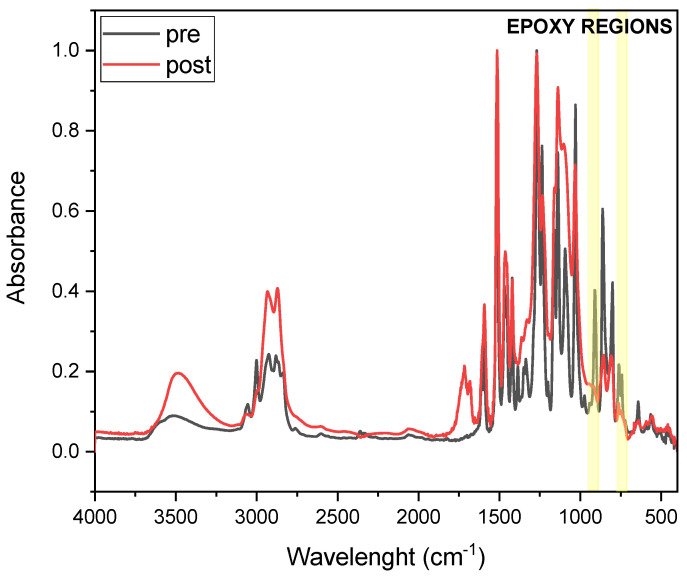
Pristine DGEVA FTIR spectra pre and post thermal curing (Yellow highlight is the region followed for epoxy group conversion).

**Figure 6:**
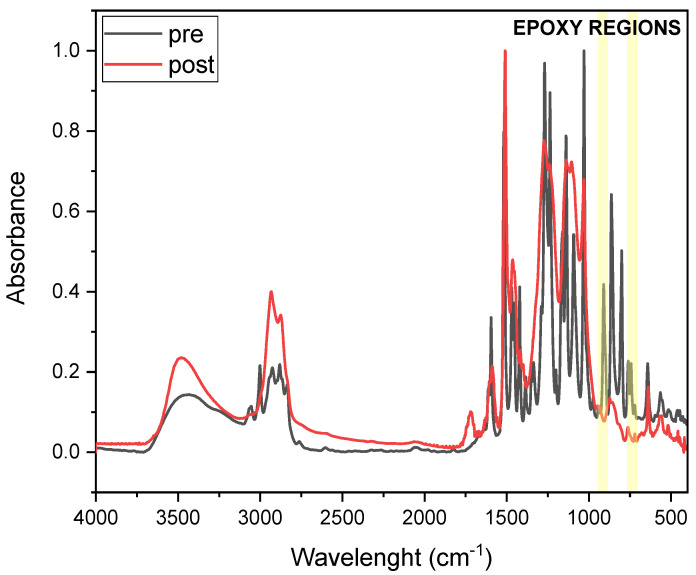
The 50 phr RCF DGEVA FTIR spectra pre and post thermal curing (Yellow highlight is the region followed for epoxy group conversion).

FTIR analysis was conducted to investigate epoxy group conversion upon thermal curing of DGEVA-based formulations. Figure 5 and Figure 6 present the FTIR spectra of pristine and 50 phr RCF DGEVA, both in their uncured and cured states. The epoxy-related absorption bands are highlighted in the spectra to emphasize the key changes associated with the curing process. Table 2 shows the conversion values of the pristine resin and with 50 phr RCF and MW, to compare unfilled vs. filled formulations in terms of curing efficiency.

In the case of pristine DGEVA, the pre-cured spectrum exhibits characteristic epoxy signals in the region around 915–830 cm^−1^, corresponding to the oxirane ring vibrations. After thermal curing, a significant reduction in these peaks is observed, indicating the successful consumption of epoxy groups during network formation. Concurrently, an increase in absorbance around 1100–1200 cm^−1^ suggests the formation of ether linkages, further confirming the progression of the crosslinking reaction.

For the DGEVA formulation containing 50 phr RCF, a similar trend is observed, with a noticeable decrease in epoxy-related peaks upon curing. However, a comparison with pristine formulation reveals some differences. The post-cured spectrum of the RCF filled sample exhibits lower residual intensity in the epoxy region with respect with the pristine one, suggesting higher conversion of oxirane rings. This could be attributed to the presence of carbon fiber reinforcement, which may increase the thermal conductivity and heat transfer or catalytic effects, thereby influencing the curing process. Additionally, slight shifts in some spectral bands indicate possible interactions between the polymer matrix and the RCF filler, potentially modifying the local curing environment.

On the other hand, a different trend is observed for the MW-filled formulation. In this case, conversion is reduced with respect to the pristine formulation, indicating a potential chain mobility restriction that leads to a less efficient process.

Overall, the FTIR results confirm the effective crosslinking of DGEVA in both the pristine and filled formulations, while also highlighting the beneficial impact of RCF and the detrimental effect of MW on the extent of epoxy conversion.

### 3.2. Dynamic DSC and DSC of Cured Samples

**Figure 7:**
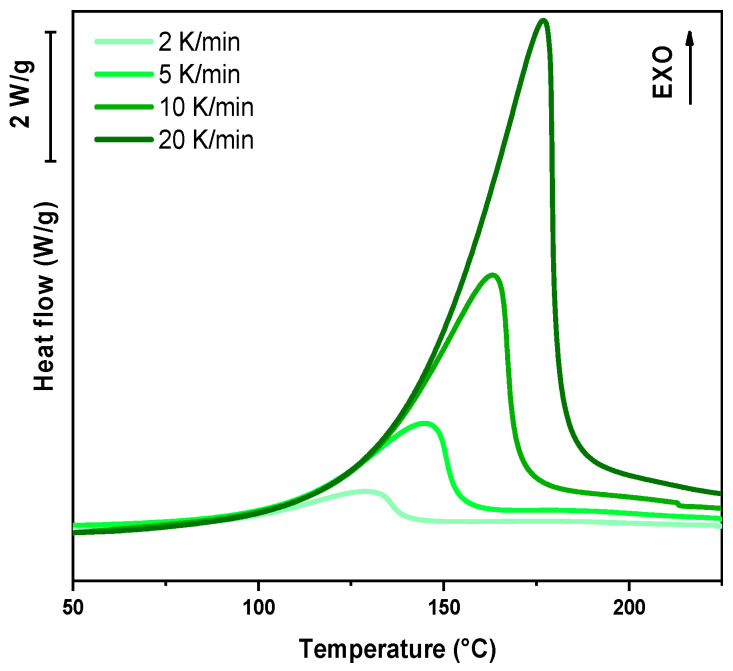
DSC analysis of pristine formulation at different heating rates.

**Figure 8:**
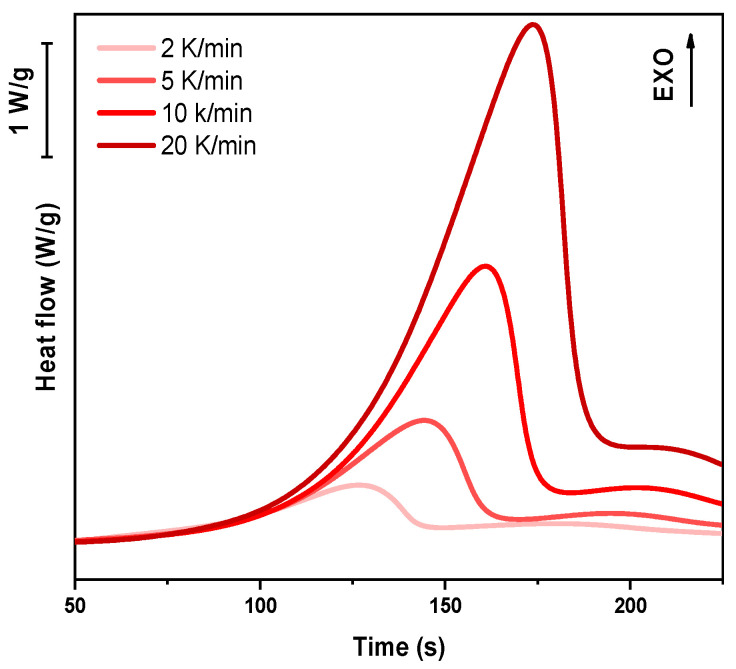
DSC analysis of DGEVA with 10 phr RCF at different heating rates.

**Figure 9:**
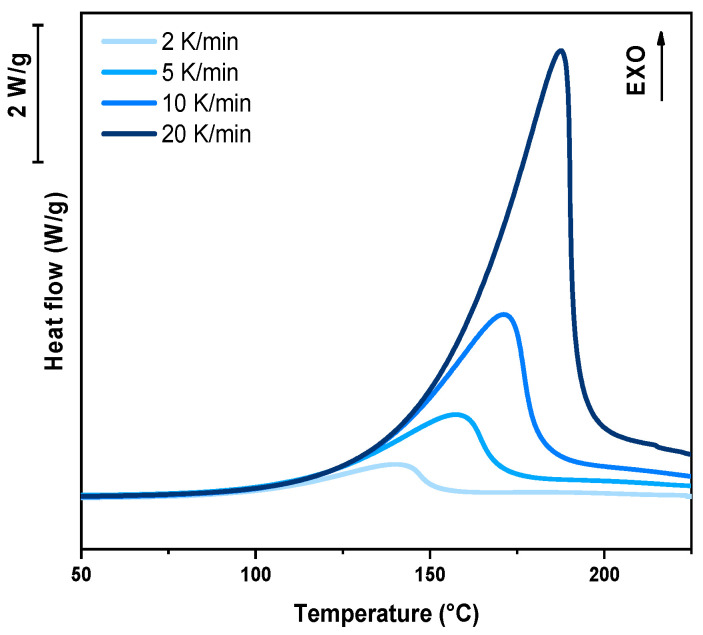
DSC analysis of DGEVA with 10 phr MW at different heating rates.

**Figure 10:**
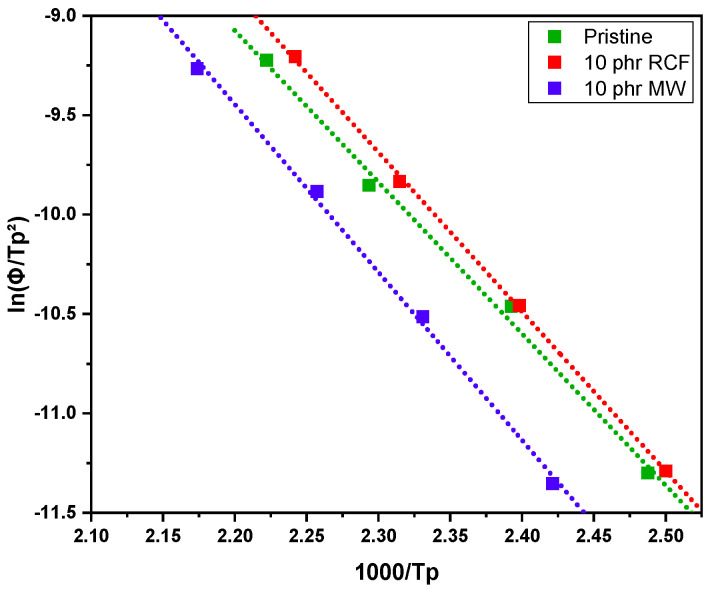
Kissinger plot of pristine DGEVA and with 10 phr of RCF and MW.

In the following paragraph, the results of dynamic DSC analysis for DGEVA-based formulations are reported in Figure 7, Figure 8 and Figure 9, and the data are collected in Table 3. In addition, the Kissinger plot is shown in Figure 10 for pristine and 10 phr formulations.

The dynamic DSC data and the resulting Kissinger analysis provide insights into the curing behavior of the pristine and filled formulations. The peak polymerization temperature (T_p_) of the pristine system is found at 163 °C, with an associated activation energy (E_a_) of 63 kJ/mol. The incorporation of 10 phr RCF leads to a slight decrease in T_p_ to 159 °C, coupled with an increase in E_a_ to 67 kJ/mol. This suggests that the presence of RCF may facilitate the initiation of the reaction at lower temperatures, potentially due to its influence on heat transfer or catalytic effects, while simultaneously requiring higher energy to sustain the overall polymerization process.

Conversely, the addition of 10 phr MW results in an elevated T_p_ of 170 °C and a further increase in E_a_ to 70 kJ/mol. This shift indicates that the MW filler potentially restricts chain mobility or introduces interactions that delay the onset of polymerization, thereby demanding a higher activation energy. Such behavior may be attributed to enhanced physical or chemical interactions between the MW filler and the reactive species, leading to a modified reaction pathway, in accordance with FTIR analysis.

**Figure 11:**
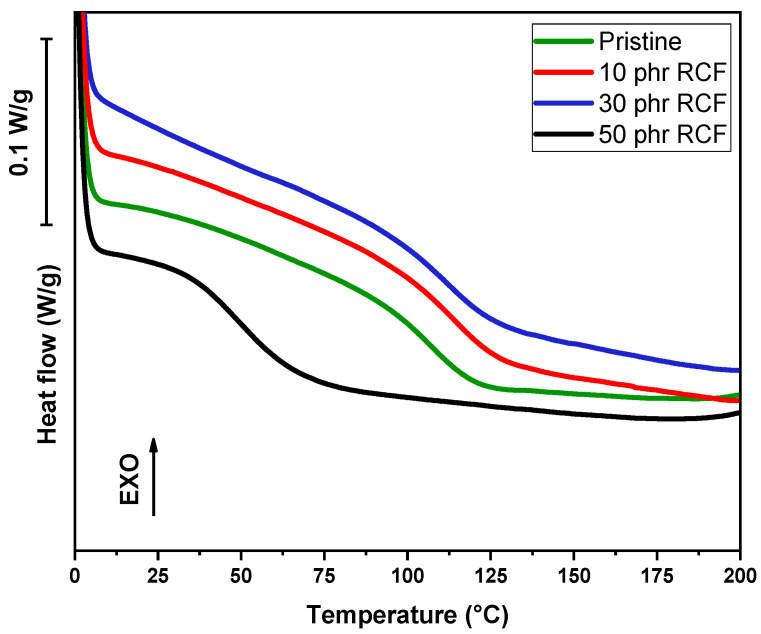
DSC curves of pristine DGEVA and with different RCF amounts.

**Figure 12:**
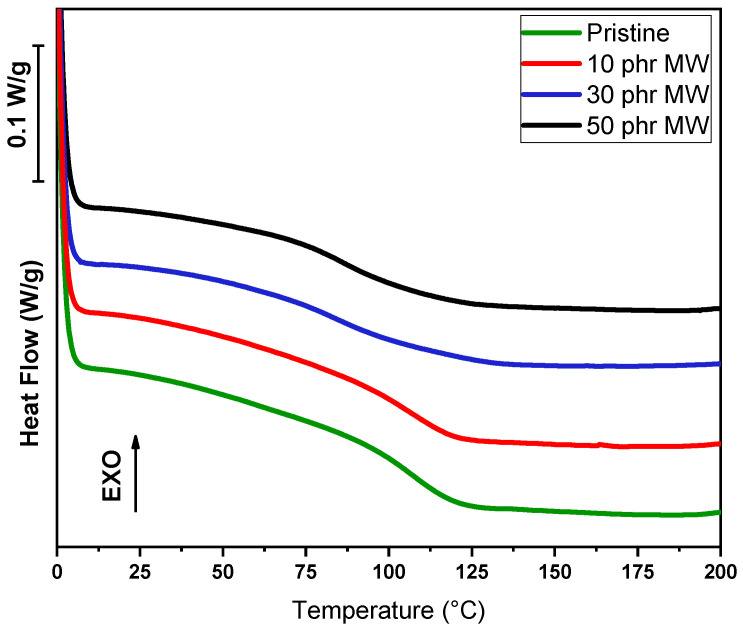
DSC curves of pristine DGEVA and with different MW content.

In addition to dynamic DSC conducted on liquid formulations, DSC analysis was conducted on cured samples, in order to assess the glass transition temperature value (Tg), as visible in Figure 11 and Figure 12 and in Table 4.

**Figure 13:**
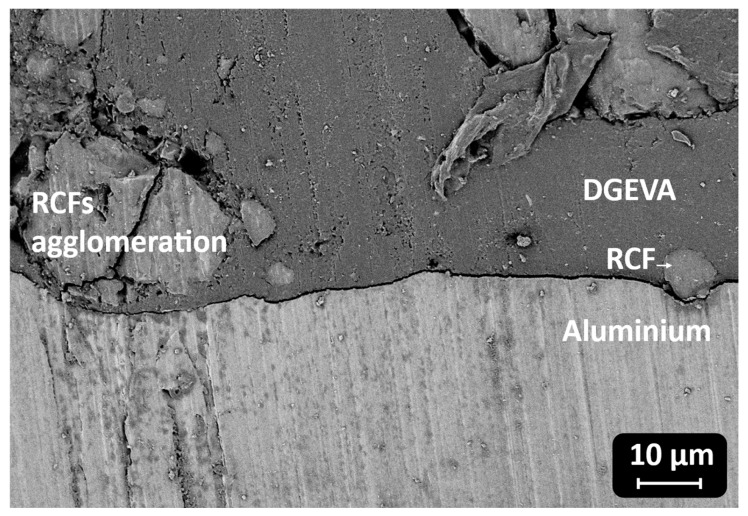
Filler agglomeration at the interface with the substrate.

The DSC analysis of the samples reveals significant variations in the Tg depending on the type and amount of filler incorporated into the polymer matrix. The pristine sample, serving as a reference, exhibits a Tg of 107 °C. Upon the addition of RCF at 10 phr, an increase in Tg to 114 °C is observed, suggesting enhanced polymer–filler interactions that restrict the mobility of polymer chains. However, at 30 phr, Tg slightly decreases to 112 °C, which may indicate a saturation effect where additional filler no longer reinforces the matrix to the same extent. A more pronounced drop is detected at 50 phr, where Tg falls drastically to 49 °C. This unexpected decrease suggests potential phase separation, filler agglomeration, or plasticization effects, leading to a reduction in the overall rigidity of the material, as visible in Figure 13.

A different trend is observed with the incorporation of MW. At 10 phr, Tg remains unchanged at 107 °C, implying minimal interaction at this filler concentration. As the filler content increases to 30 phr, Tg drops significantly to 85 °C, suggesting a plasticization effect or poor compatibility between the filler and the polymer matrix, which disrupts the polymer network. At 50 phr, a slight recovery is observed, with Tg increasing to 86 °C. This suggests that at higher loadings, some degree of network stabilization might occur, although the glass transition temperature remains considerably lower than that of the pristine sample.

The contrasting behaviors observed for RCF and MW fillers indicate differences in their interactions with the polymer matrix. While low concentrations of RCF appear to reinforce the material, excessive amounts likely lead to agglomeration, negatively impacting thermal properties. The poor interfacial adhesion, due to filler agglomeration, is responsible for a poorer stress transfer between the recycled carbon fibers and the resin matrix, and it will induce a decrease in thermos-mechanical properties of the crosslinked adhesive. This morphological feature may explain the lower mechanical properties observed for the corresponding samples, better discussed in Section 3.4., Shear Lap Test.

Conversely, by increasing the MW filler, it predominantly acts as a plasticizer, reducing Tg across all concentrations tested.

### 3.3. DMTA

**Figure 14:**
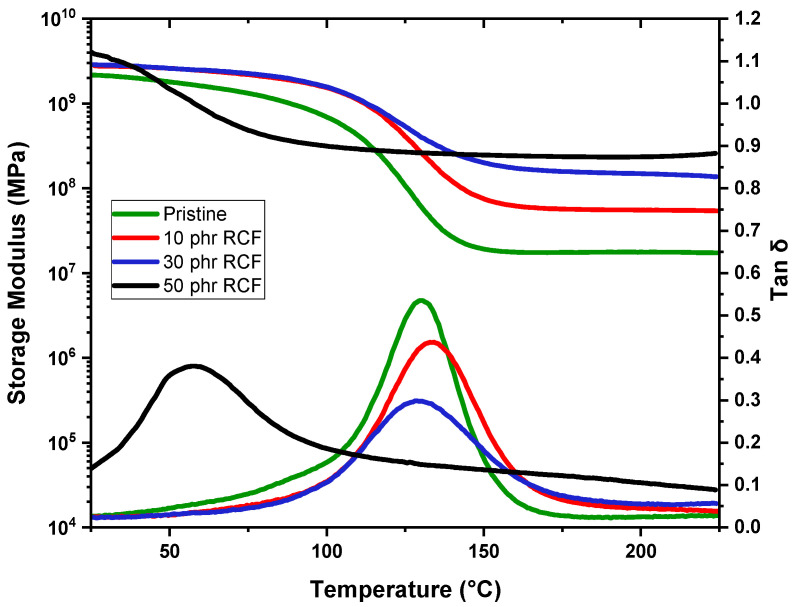
Storage modulus and Tanδ as a function of temperature of DGEVA pristine and with RCF filler.

**Figure 15:**
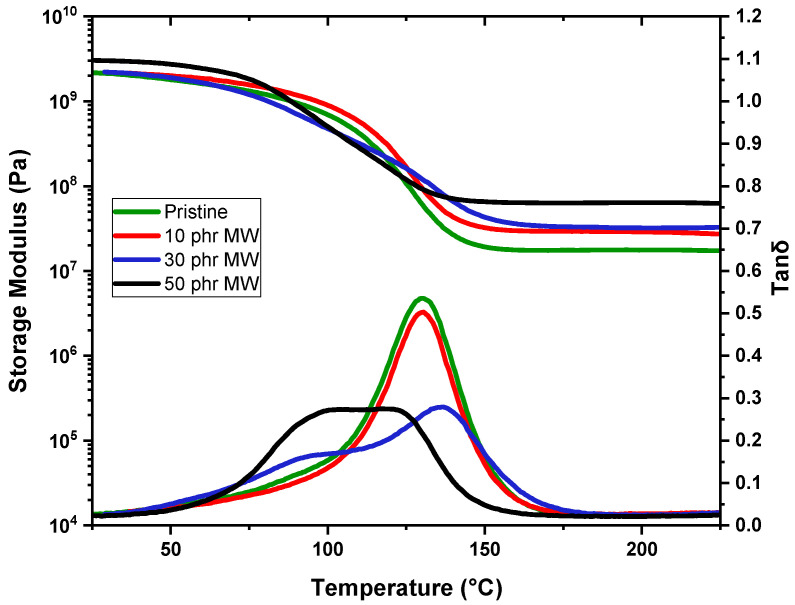
Storage modulus and Tanδ as a function of temperature of DGEVA pristine and with MW filler.

In the following paragraph, DMTA results are reported in Figure 14 and Figure 15, and in Table 5 for the crosslinked formulations.

DMTA results provide further insights into the glass transition behavior of the studied samples, complementing the trends observed in the DSC data. The pristine sample exhibits a Tg of 131 °C, serving as the reference for comparison. Upon the addition of RCFs at 10 phr, Tg increases slightly to 134 °C, confirming the previously observed reinforcing effect, where the filler restricts polymer chain mobility, leading to an elevation in Tg. However, at 30 phr, Tg decreases to 125 °C, which aligns with the DSC trend indicating a saturation effect, where additional filler does not contribute as effectively to matrix stiffening. The most striking behavior occurs at 50 phr, where Tg drops significantly to 59 °C. This drastic reduction, also observed in DSC, suggests severe phase separation or plasticization effects due to filler aggregation, disrupting the polymer network and reducing the effectiveness of polymer–filler interactions.

The MW-filled samples exhibit a different trend. At 10 phr, Tg remains nearly unchanged at 130 °C, like the pristine material, indicating minimal interaction at this concentration. With increasing MW content, Tg follows an irregular pattern: at 30 phr, it decreases significantly to 96 °C, while at 50 phr, it increases again to 105 °C. The slight recovery of Tg at 50 phr suggests that at higher concentrations, MW may contribute to some degree of structural stabilization, possibly due to network percolation effects or secondary interactions reinforcing the polymer matrix.

Regarding the storage modulus in the rubbery plateau (measured at Tg + 50 °C), the pristine DGEVA exhibits a low modulus of 17 MPa, consistent with its soft polymeric state above Tg. The inclusion of RCFs significantly enhances the modulus, with values rising to 56 MPa, 155 MPa, and 285 MPa for 10, 30, and 50 phr, respectively, reflecting a strong reinforcing effect even though this is accompanied by the noted decrease in Tg at high loadings. Meanwhile, MW yields a more moderate improvement in stiffness, with storage moduli of 28 MPa, 32 MPa, and 63 MPa at 10, 30, and 50 phr, respectively.

Overall, while RCF markedly increases the mechanical stiffness of DGEVA, its high loadings adversely affect the thermal behavior, possibly limiting its applicability. Conversely, MW offers a compromise, providing moderate reinforcement with a less pronounced impact on Tg.

### 3.4. Shear Lap Test

**Figure 16:**
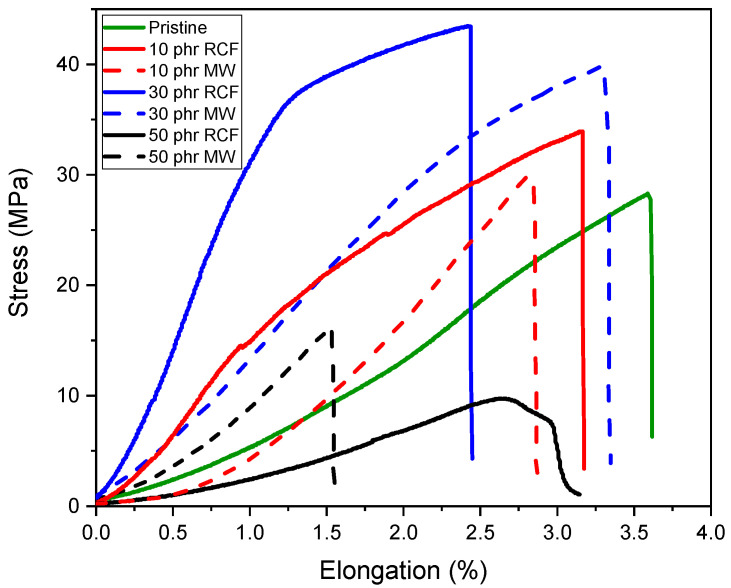
Shear lap test performed on CMC joints.

**Figure 17:**
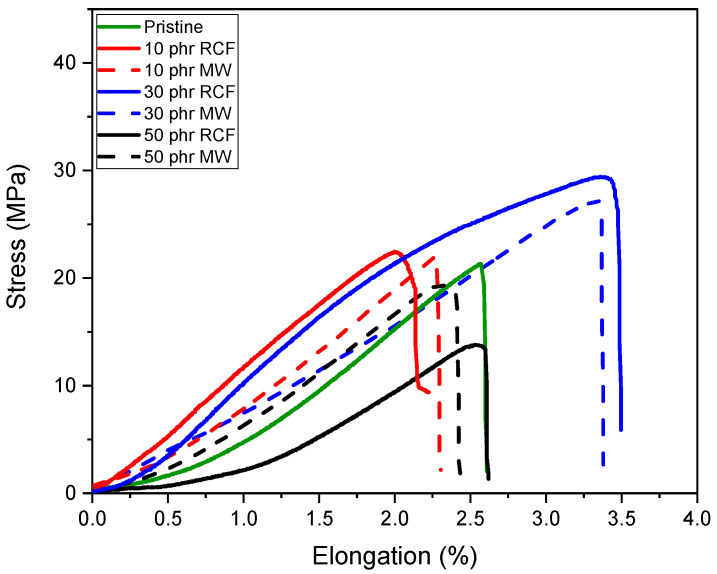
Shear lap test performed on Al joints.

**Figure 18:**
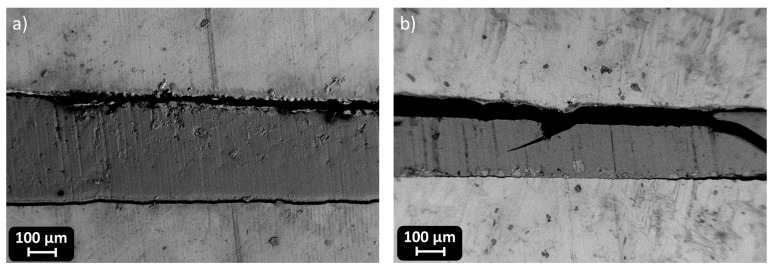
Interface of 10 phr RCF (**a**) and 10 phr MW (**b**) Al joints.

The mechanical performance of the DGEVA-based adhesive formulations, modified with varying amounts of recycled carbon fibers (RCFs) and Mineral Wool (MW), was evaluated through shear lap tests using two different substrates: Ceramic Matrix Composite (CMC) and aluminum (Al). As shown in Figure 16 and Table 6, CMC joints exhibited higher shear strengths than their Al counterparts, reported in Figure 17 and Table 7, with all CMC samples displaying cohesive failure within the adhesive layer, indicating good adhesion to the substrate. Conversely, Al joints systematically failed via adhesive rupture, pointing to weaker interfacial bonding, as visible from Figure 18.

In CMC joints, the incorporation of RCFs significantly improved the maximum shear strength, reaching a peak of 43 ± 1 MPa at 30 phr, before drastically decreasing to 10 ± 3 MPa at 50 phr, due to filler agglomeration or poor dispersion at high loadings, as already reported and visible in Figure 13. A similar trend was observed with MW, where a maximum strength of 40 ± 1 MPa was recorded at 30 phr, dropping to 15 ± 2 MPa at 50 phr. These observations correlate well with the dynamic mechanical properties. As already discussed, and reported in Table 4, the storage modulus in the rubbery plateau (E′ at Tg + 50 °C) increased markedly with filler content, particularly for RCFs at 30 and 50 phr (155 and 285 MPa, respectively), reflecting the formation of a rigid filler network. However, the pronounced drop in Tg at 50 phr RCF (59 ± 5 °C) suggests disrupted polymer network continuity, which could compromise cohesive strength despite the high stiffness.

In contrast, Al joints showed more moderate strength values, ranging from 21 ± 1 MPa for the pristine formulation to 29 ± 1 MPa at 30 phr RCF and 27 ± 1 MPa at 30 phr MW. Although filler addition improved the load-bearing capacity, the adhesive failure mode indicates that substrate–adhesive interfacial strength remained the limiting factor. Interestingly, the highest strain at break in Al joints was observed for 30 phr RCF (3.4 ± 0.2%), suggesting a good balance between stiffness and ductility at this composition. However, excessive filler addition again proved detrimental, as evidenced by the reduction in both strength and elongation at 50 phr.

## 4. Conclusions

This study presents a sustainable approach for the development of structural adhesives by formulating a fully bio-based thermosetting system based on Diglycidylether of vanillyl alcohol (DGEVA), reinforced with recycled carbon fibers (RCFs) and mineral wool (MW). The influence of filler type and content was systematically evaluated across thermal, chemical, and mechanical properties. The curing process, monitored by FTIR and DSC, confirmed effective crosslinking in all formulations, though higher filler contents generally led to slower polymerization kinetics and increased activation energy. Despite these effects, high conversion rates were achieved, ensuring the formation of a robust polymer network.

Thermal analysis highlighted a dual effect of filler addition on the glass transition temperature (Tg): while moderate RCF loading enhanced Tg, excessive filler content, especially at 50 phr, led to a dramatic decrease, indicating network disruption or phase separation. MW, on the other hand, behaved more like a plasticizer, causing a consistent reduction in Tg, particularly at intermediate loadings. DMTA confirmed these trends, with RCFs yielding a substantial increase in stiffness in the rubbery plateau region, whereas MW provided only moderate mechanical reinforcement.

Shear lap tests demonstrated the effectiveness of both fillers in improving adhesive strength, especially on Ceramic Matrix Composite (CMC) substrates, where cohesive failure dominated. Maximum strength was achieved at 30 phr for both RCFs and MW, with a marked decline at higher loadings due to filler agglomeration or reduced matrix integrity. On aluminum substrates, where adhesive failure occurred in all cases, the improvements were less pronounced, suggesting limited interaction between the adhesive and the metal surface.

In conclusion, the developed DGEVA-based adhesives reinforced with recycled carbon fibers (RCFs) and mineral wool (MW) exhibit mechanical and thermal properties suitable for semi-structural bonding applications where sustainability and performance must coexist. Their high shear strength on ceramic matrix composites (up to 43 MPa), combined with enhanced stiffness and thermal resistance, makes them promising candidates for lightweight composite joining and repair in automotive, transportation, and aerospace interior components, where reduced environmental impact and recyclability are increasingly prioritized [1,3,14,19].

In addition, the compatibility with mineral-based fillers supports their use in construction and building materials, such as bonding of insulation panels, facade laminates, or eco-composite elements, contributing to waste valorization of demolition residues. The moderate glass transition temperature of DGEVA also enables potential use in electronic housings, fixtures, and protective coatings that require stable adhesion under thermal cycling. Overall, these adhesives combine mechanical efficiency, recyclability, and resource circularity, offering a sustainable alternative to petroleum-based structural bonding systems.

## Figures and Tables

**Table 1:** List of formulations analyzed.

Amount	Type	
0 phr	DGEVA pristine	
10 phr	RCF	MW
30 phr	RCF	MW
50 phr	RCF	MW

**Table 2:** Conversion values of pristine, 50 phr RCF and MW formulations.

Type	Conversion
Pristine	92 ± 1%
50 phr RCF	95 ± 1%
50 phr MW	90 ± 1%

**Table 3:** Values of peak temperature (Tp) and activation energy (Ea) of the studied DGEVA-based formulations.

Filler	T_p_ [°C]	E_a_ [KJ/mol]
Pristine	163	63
10 phr RCF	159	67
10 phr MW	170	70

**Table 4:** Tg values of the samples studied.

Filler	Amount [phr]	Tg [°C]
Pristine	0	107
RCF	10	114
	30	112
	50	49
MW	10	107
	30	85
	50	86

**Table 5:** Tg and storage modulus in the rubbery plateau values of the studied samples.

Fillers	Amount [phr]	Tg [°C]	E’(Tg + 50 °C) [MPa]
Pristine	0	131 ± 1	17 ± 2
RCF	10	134 ± 1	56 ± 1
	30	125 ± 3	155 ± 1
	50	59 ± 5	285 ± 4
MW	10	130 ± 1	28 ± 1
	30	96 ± 2	32 ± 2
	50	105 ± 1	63 ± 1

**Table 6:** Average of stress and elongation at break of the analyzed CMC joints.

Filler	Amount	Max Stress [Mpa]	Strain at Break [%]
Pristine	No filler	27 ± 2	3.6 ± 0.6
RCF	10 phr	34 ±2	3.2 ± 0.5
	30 phr	43 ±1	2.4 ± 0.2
	50 phr	10 ± 3	2.8 ± 0.6
MW	10 phr	30 ± 4	2.9 ± 0.5
	30 phr	40 ± 1	3.3 ± 0.2
	50 phr	15 ± 2	1.5 ± 0.3

**Table 7:** Average of stress and elongation at break of the analyzed Al joints.

Filler	Amount	Max Stress [Mpa]	Strain at Break [%]
Pristine	No filler	21 ± 1	2.5 ± 0.3
RCF	10 phr	23 ± 1	2.0 ± 0.2
	30 phr	29 ± 1	3.4 ± 0.2
	50 phr	14 ± 2	2.6 ± 0.3
MW	10 phr	22 ± 1	2.3 ± 0.2
	30 phr	27 ± 1	3.3 ± 0.1
	50 phr	20 ± 2	2.4 ± 0.1

## Data Availability

The original contributions presented in this study are included in the article. Further inquiries can be directed to the corresponding author.
